# PLAC8 participates in the prognosis regulation of breast cancer by regulating the infiltration of immune cells in the tumor microenvironment

**DOI:** 10.3389/fbinf.2026.1814907

**Published:** 2026-08-14

**Authors:** Mengqi Yu

**Affiliations:** School of Pharmacy and Pharmaceutical Sciences, Cardiff University, Cardiff, United Kingdom

**Keywords:** breast cancer, mechanism, tumor microenvironment, biomarkers, PLAC8, immunotherapy, prognosis

## Abstract

**Objective:**

To investigate the mechanism of Placenta-specific 8 (PLAC8) in the tumor microenvironment (TME) of breast cancer.

**Methods:**

Two single-cell RNA-seq datasets (GSE161529 for primary breast cancer, GSE150660 for leptomeningeal metastasis) were integrated to profile microenvironmental composition and PLAC8 expression. TISCH2 was used for cell annotation, differential expression and transcription factor enrichment of PLAC8-positive immune clusters. Tumor purity-adjusted partial correlation, survival Cox regression and immune stratified prognosis analyses based on TCGA-BRCA were conducted via TIMER. GeneMANIA and Sangerbox were adopted to construct PLAC8-centered protein interaction networks and perform Gene Ontology functional enrichment.

**Results:**

Primary and metastatic lesions displayed drastically remodeled immune landscapes, with monocytes/macrophages and CD8^+^ T cells dominating metastatic immune compartments. PLAC8 was specifically highly expressed in tumor-infiltrating B cells, whereas malignant epithelial cells barely transcribed PLAC8 in both cohorts. PLAC8 positively correlated with cell cycle/stress transcription factors (PML, E2F4, MYC) and negatively correlated with epithelial/estrogen regulators (FOXA1, ESR1). Cox analyses verified PLAC8 upregulation as an independent favorable prognostic marker. Distinct immune-dependent prognostic patterns were observed: NKT cell protective effects were PLAC8-independent; CD8^+^ central memory T cell survival benefits relied on PLAC8-modulated immune networks; memory B cells and pDCs only exerted anti-tumor protective effects in high-PLAC8 tumors. Enrichment results indicated PLAC8-related transcriptional and biosynthetic programs mainly support immune cell effector activation rather than malignant progression of tumor cells.

**Conclusion:**

This integrated single-cell analysis identifies B-cell-specific enrichment of PLAC8 in breast cancer TME for the first time. PLAC8 reshapes immune homeostasis via core transcriptional networks and mediates heterogeneous immune synergistic prognostic effects. These findings provide novel mechanistic insights into breast cancer immune heterogeneity and candidate biomarkers for clinical prognosis stratification and immunotherapy research.

## 1 Introduction

Breast cancer is the most common malignancy in women, with approximately 2.3 million new cases reported worldwide in 2022, accounting for 11.6% of all cancers (Bray et al., 2024). During breast cancer progression, the tumor microenvironment plays a pivotal regulatory role, primarily by sculpting immune responses to enable immune escape and by reprogramming stromal cells to adopt pro-tumorigenic functions (Chew et al., 2012). In recent years, the application of single-cell RNA sequencing (scRNA-seq) has illuminated the profound cellular heterogeneity underlying cancer metastasis, catalyzing major shifts in the field and providing critical insights into the metastatic mechanisms of breast cancer at cellular resolution (Liu et al., 2024; Yang et al., 2024).

PLAC8, located on human chromosome 4 and classified as a member of the keratinizing epithelium protein family, was originally identified as a placenta-associated regulatory gene (Mao et al., 2021a). Existing research reveals that PLAC8 displays bifunctional biological activities in a range of malignancies:it acts as an oncogenic driver in lung, colorectal, and prostate cancers by enhancing tumor cell proliferation (Kolluru et al., 2017; Huang et al., 2020; Chen et al., 2022a), whereas it demonstrates a pronounced tumor-suppressive role in hepatocellular carcinoma (Zou et al., 2016). In breast cancer, PLAC8 promotes tumor growth by activating the PI3K/AKT/NF-κB signaling cascade and suppressing apoptosis (Mao et al., 2019), and it has also been implicated in the development of multidrug resistance (Mao et al., 2021b). In triple-negative breast cancer, PLAC8 is generally expressed at high levels and can modulate PD-L1 ubiquitination, thereby influencing tumor cell proliferation and antitumor immune responses (Mao et al., 2022). Despite these findings, the precise mechanistic actions of PLAC8 in breast cancer and its impact on the tumor microenvironment require further in-depth investigation. In this study, we integrated single-cell RNA-sequencing datasets of breast cancer (GSE161529 and GSE150660) to systematically characterize TME cellular composition and to explore the immunological infiltration patterns of PLAC8 in relation to patient prognosis. Through multi-layered bioinformatic analyses, we aim to elucidate how PLAC8 shapes breast cancer progression by regulating transcriptional programs, immune cell functionality, immune responses, and key signaling pathways, thereby providing theoretical support for prognostic assessment and precision immunotherapy.

## 2 Materials and methods

### 2.1 Data sources and clinical characteristics

This study incorporated two publicly available single-cell transcriptomic datasets from the high-throughput gene expression repository. (1) GSE161529 comprises 332,168 cells derived from 33 patients with primary breast cancer, including 4 triple-negative breast cancer (TNBC) cases, 4 BRCA1-mutated TNBC cases, 6 HER2-positive tumors, and 19 estrogen receptor–positive (ER+) tumors. (2) GSE150660 contains 10,605 cells obtained from three patients diagnosed with leptomeningeal metastases of breast cancer. All samples were generated using the 10x Genomics single-cell RNA sequencing platform.

### 2.2 Single-cell data analysis platforms

#### 2.2.1 TISCH2 platform

The TISCH2 platform (http://tisch.comp-genomics.org/) is an integrated public resource compiling standardized single-cell RNA-sequencing (scRNA-seq) datasets derived from human and murine tumor specimens. This database supports multi-layer tumor microenvironment exploration and integrates multiple analytical modules, including pre-generated cell-type annotation pipelines, inter-cluster differential expression calculation, LISA-based transcription factor (TF) enrichment prediction, cell-cell interaction inference and gene expression visualization, with detailed sample metadata embedded for all deposited cohorts (Han et al., 2022).

Two public GEO single-cell datasets (GSE161529 and GSE150660) were uniformly preprocessed, batch-normalized and assigned cell labels via the built-in MAESTRO pipeline of TISCH2. All downstream single-cell analyses in this work were performed on the standardized expression matrices and cell annotations generated automatically by the platform.

Two core analytical workflows embedded in TISCH2 were implemented for PLAC8-related transcriptional characterization: inter-cluster differential expression analysis and LISA chromatin-based TF enrichment prediction. All analytical operations were performed at single-cell resolution, with individual cell treated as the basic analysis unit. To identify PLAC8-expressing immune clusters, the official TISCH2 threshold criteria were applied: clusters with log_2_ (fold change) > 0 were screened as candidate high-expression groups, and only clusters with Benjamini–Hochberg adjusted p < 0.05 were retained as PLAC8-high immune subpopulations for subsequent TF enrichment. The LISA model was exclusively run on these qualified PLAC8-high monocyte and macrophage clusters to screen tissue-specific transcriptional regulators supported by human breast epigenetic datasets.

#### 2.2.2 TIMER platform

The TIMER platform (http://timer.cistrome.org/) is a comprehensive analytical tool designed to systematically evaluate immune infiltration patterns across various cancer types. It integrates multiple state-of-the-art algorithms to estimate immune cell infiltration and enables exploration of associations among immune infiltration levels, gene expression, somatic mutations, and clinical outcomes within TCGA cohorts.

In this study, TIMER was employed to assess the relationships between PLAC8 expression and key immune-related genes as well as immune-infiltrating cell subsets in breast cancer. The platform was further used to examine the associations among PLAC8 expression, the degree of immune cell infiltration, and clinical outcomes in breast cancer patients.

#### 2.2.3 GeneMANIA platform

The GeneMANIA database (https://genemania.org/) is an online tool for gene function prediction and network analysis. It constructs multi-dimensional functional interaction networks by integrating weighted co-expression analysis (Pearson correlation coefficient >0.85), physical interaction data from the STRING database (version 11.5), and genetic association evidence from the GWAS Catalog (FDR <0.05). The resulting networks encompass protein–protein interactions, genetic interactions, co-expression, co-localization, protein domain similarity, and shared pathway features (Warde-Farley et al., 2010).

In the present study, GeneMANIA was applied to construct a multidimensional functional interaction network centered on PLAC8, to screen functionally linked partner genes with close regulatory correlation. All candidate genes output from the GeneMANIA network were collected as the input gene set for subsequent Gene Ontology enrichment analysis.

#### 2.2.4 Sangerbox platform

Sangerbox 3.0 (https://sangerbox.com/) is an integrated online bioinformatics analysis platform supporting gene set enrichment and multi-omics visualization. Gene Ontology (GO) functional enrichment analysis was performed on the candidate gene set derived from GeneMANIA using the built-in enrichment pipeline of Sangerbox 3.0.

The enrichment workflow relied on R package clusterProfiler (version 3.14.3) and human gene annotation database org. Hs.e.g.,.db (version 3.1.0). All human protein-coding genes were set as the universal background gene set. The filtering thresholds for gene sets were set as minimum gene count = 5 and maximum gene count = 5,000. Two statistical thresholds were defined for significant enrichment results: P < 0.05 and FDR <0.25. FDR was calculated for each GO term to correct multiple testing bias.

All enriched biological process terms were ranked by -log_10_(*P*) value, and the top enriched terms were visualized for result presentation. Full statistical data including adjusted FDR values of all GO entries were exported for supplementary tables.

### 2.3 Statistical analysis

All statistical tests conducted on single-cell datasets via the TISCH2 platform followed standard built-in pipelines. Inter-cluster differential expression was assessed with Wilcoxon rank-sum tests, and the Benjamini–Hochberg procedure was applied to correct multiple testing bias. The LISA transcription factor enrichment model relied on hypergeometric testing, with a fixed background set of 3,000 randomly sampled human protein-coding genes. Log_2_ (fold change) and adjusted *P*-values provided by TISCH2 were adopted to screen PLAC8-high immune cell clusters.

Bulk-level correlation and survival analyses for TCGA-BRCA cohort were processed on the TIMER platform. Samples lacking complete gene expression or immune infiltration metadata were automatically excluded by the platform workflow, leaving 1,093–1,100 primary invasive breast carcinoma cases for downstream analysis. All correlation evaluations adopted partial Spearman’s correlation adjusted for tumor purity to eliminate confounding bias derived from heterogeneous immune and stromal cell proportions; all visualized correlation outputs were generated under this correction framework. Survival stratification was performed based on PLAC8 expression and immune infiltration levels. Kaplan–Meier curves were plotted to compare overall survival disparities, with log-rank tests for inter-group significance. Cox proportional hazards regression models were used to calculate hazard ratios and 95% confidence intervals.

Gene Ontology enrichment statistics were calculated on the Sangerbox platform. Gene sets with sizes ranging from 5 to 5,000 were retained for enrichment computation. Statistical significance was defined as *P* < 0.05 and FDR <0.25, and FDR values were calculated for each term to offset multiple testing errors.

For all analytical procedures in this study, two-tailed *P* < 0.05 was defined as the threshold of statistical significance.

All analyses were conducted via the official web interfaces of four public platforms (TISCH2, TIMER2.0, GeneMANIA, Sangerbox 3.0). No custom local R/Python scripts were used. All statistical pipelines and parameters followed the built-in default workflows documented in database references. Raw output tables with full statistics are supplied as Supplementary Material for repeatability.

## 3 Results

### 3.1 Cellular composition of the breast cancer tumor microenvironment

**FIGURE 1:**
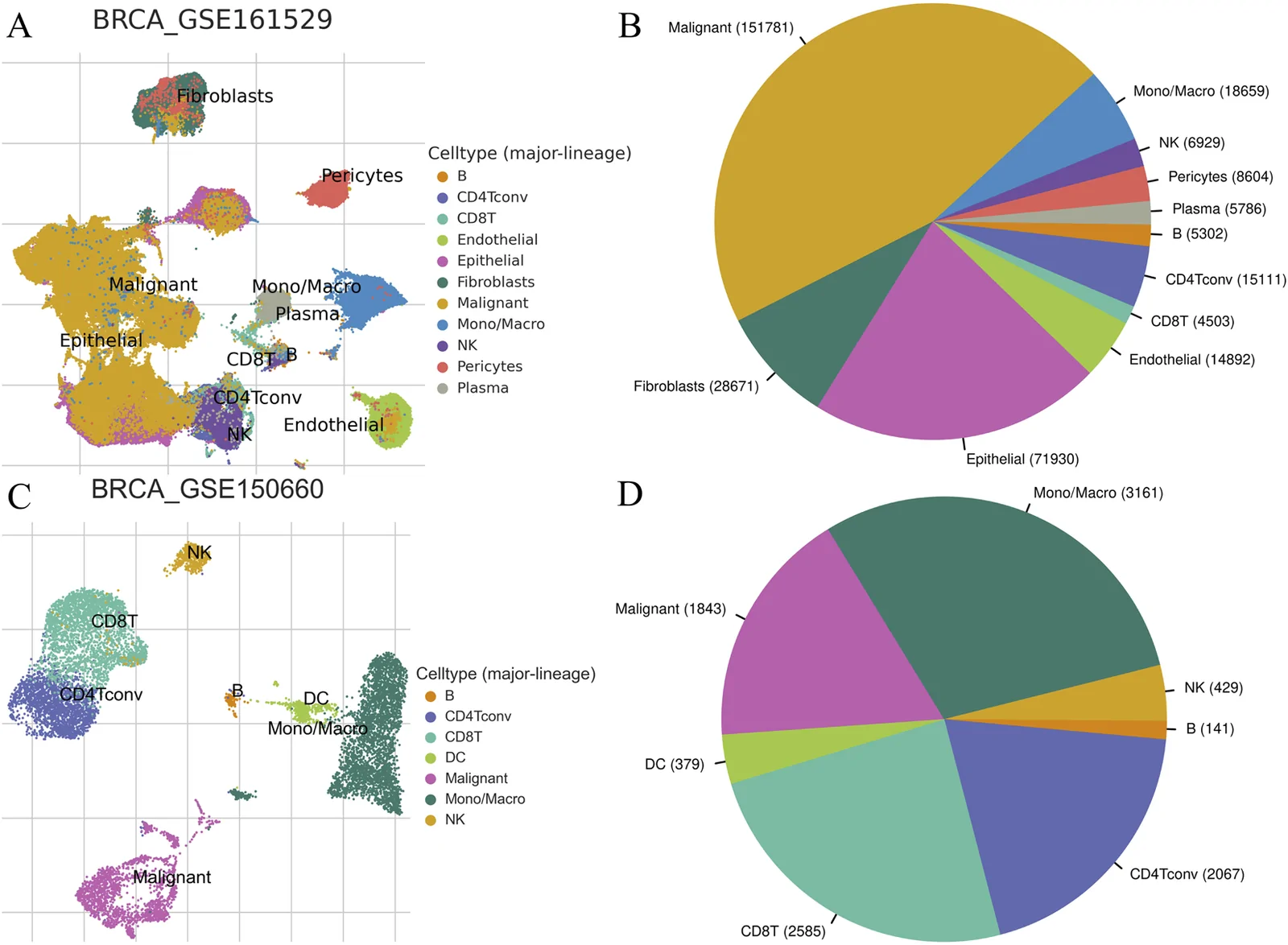
Single-cell transcriptomic profiling of the breast cancer tumor microenvironment reveals distinct immune landscapes between primary and metastatic lesions: **(A)** Visualization of single-cell RNA-sequencing data from primary breast cancer tissues. **(B)** Cellular composition of the tumor microenvironment in primary breast cancer. **(C)** Visualization of single-cell RNA-sequencing data from metastatic breast cancer (leptomeningeal metastasis). **(D)** Immune microenvironment composition in metastatic breast cancer.

Cell-type annotation was directly adopted from the pre-annotated cell classification results embedded in the TISCH2 database. In the primary breast cancer dataset (GSE161529), six immune cell subsets (B cells, CD4^+^ T cells, CD8^+^ T cells, NK cells, monocytes/macrophages, and plasma cells), five stromal cell types (endothelial cells, epithelial cells, fibroblasts and pericytes) and malignant cells were annotated in the database (Figure 1A). Stromal cells accounted for 37.36% of all cells (124,097/332,168), while malignant cells accounted for 45.69% (151,781/332,168), and immune cells accounted for 16.95% (56,290/332,168). Immune infiltration was primarily composed of monocytes/macrophages (5.62%, 18,657/332,168) and conventional CD4^+^ T cells (4.55%, 15,111/332,168), whereas B cells (1.66%, 5,302/332,168) and CD8^+^ T cells (1.36%, 4,503/332,168) showed the lowest infiltration levels (Figure 1B).

Compared with primary tumors, the metastatic breast cancer dataset (GSE150660) exhibited substantial remodeling of immune cell composition. Monocytes/macrophages (29.06%, 3,161/10,605) and CD8^+^ T cells (23.76%, 2,585/10,605) emerged as the predominant subsets (Figure 1C). The proportion of conventional CD4^+^ T cells increased to 19% (2,067/10,605), and a small population of dendritic cells (3.57%, 379/10,605) was detected. B cells remained the least abundant immune population (1.33%, 141/10,605). In metastatic breast cancer, no *bona fide* stromal cells were observed; only malignant cells (16.94%, 1,843/10,605) were present within the non-immune compartment (Figure 1D). This implies that tumor cells at metastatic sites exhibit enhanced potential for microenvironmental remodeling.

### 3.2 Cell type–specific expression patterns of PLAC8 in the TME

We next explored the cell-type-specific expression of PLAC8 in the breast cancer tumor microenvironment using single-cell transcriptomic profiles from primary (GSE161529) and metastatic (GSE150660) specimens.

**FIGURE 2:**
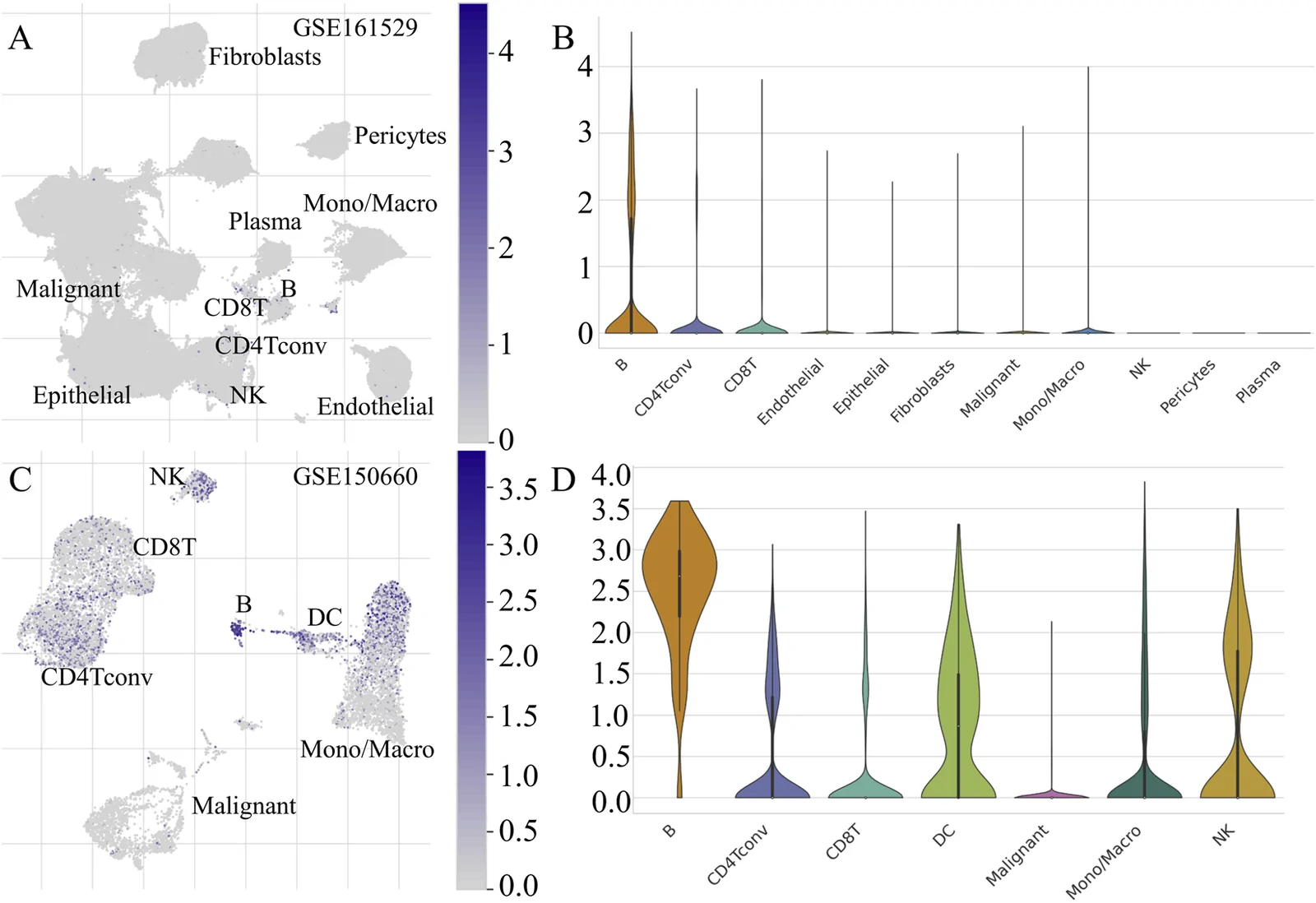
Cell type–specific expression profiles of PLAC8 in the breast cancer tumor microenvironment. **(A)** UMAP visualization of PLAC8 expression across all cell types in primary breast cancer (GSE161529). **(B)** Violin plots quantifying PLAC8 expression levels in distinct cellular subsets from primary breast cancer. **(C)** UMAP visualization of PLAC8 expression across all cell types in metastatic breast cancer (GSE150660). **(D)** Violin plots quantifying PLAC8 expression levels in distinct cellular subsets from metastatic breast cancer.

In primary breast cancer, UMAP visualization (Figure 2A) showed that PLAC8 expression was largely restricted to immune cell subsets, with the strongest and specific expression exclusively detected in B cells. Different from B cells, NK cells, CD4^+^ T cells, and monocytes/macrophages exhibited only low baseline PLAC8 expression, whereas malignant epithelial cells, fibroblasts, and endothelial cells showed minimal signal. Violin plot quantification (Figure 2B) confirmed that B cells displayed the highest mean PLAC8 expression (0.97), which was significantly greater than in all other immune and non-immune subsets (*P* < 0.01). NK cells, CD4^+^ T cells, and monocytes/macrophages exhibited relatively low baseline expression (0.27, 0.23, and 0.22, respectively), while malignant epithelial cells showed almost undetectable levels.

In metastatic breast cancer, UMAP analysis (Figure 2C) revealed a similar immune-dominant pattern, with PLAC8 expression again concentrated in B cells at levels substantially higher than in primary tumors. Supporting this observation, violin plots (Figure 2D) demonstrated that B cells exhibited the highest mean PLAC8 expression (3.68), followed by dendritic cells (1.25) and NK cells (1.14). Malignant cells again displayed extremely low PLAC8 expression (0.03), consistent with findings in primary disease.

Consistent single-cell profiling revealed that PLAC8 expression was predominantly restricted to B cells in both primary and metastatic breast cancer. Of note, the average PLAC8 expression level in B cells was markedly higher in metastatic leptomeningeal lesions than that in primary breast tumors.

Given that the primary and metastatic datasets were obtained from independent patient cohorts and different tissue microenvironments, and the metastatic sample size is limited, this study mainly focuses on describing the transcriptional expression pattern of PLAC8. The elevated PLAC8 level in metastatic B cells represents a preliminary observational trend. Further validation with paired primary-metastatic samples and functional experiments will be required to clarify its potential role in tumor metastatic progression and immune microenvironment remodeling.

### 3.3 Co-expression network and transcriptional regulatory features of PLAC8

To systematically dissect the transcriptional regulatory network linked to PLAC8, single-cell differential expression profiling and LISA transcription factor (TF) enrichment analysis were conducted on primary breast cancer dataset GSE161529 and metastatic breast cancer dataset GSE150660 via the public TISCH2 single-cell RNA-seq database, with bulk transcriptome co-expression analysis performed based on the two cohorts. Following the official analytical criteria of TISCH2, cell clusters with log_2_ (fold change) > 0 and Benjamini–Hochberg adjusted *p* < 0.05 were identified as PLAC8-high immune subpopulations for subsequent analysis.

For the primary dataset GSE161529, PLAC8 was highly expressed in four immune subclusters: Cluster 14 (monocytes/macrophages), Cluster 16 (NK cells), Cluster 23 (B cells) and Cluster 34 (monocytes/macrophages). Breast-specific TF enrichment revealed that monocyte/macrophage Cluster 34 was enriched with MAX, NR2F2, CTCF, ERG and MYC, B cell Cluster 23 contained KDM5B and MBD2, NK cell Cluster 16 was marked by BRCA1, and monocyte/macrophage Cluster 14 exhibited enrichment of TP53.

In the metastatic dataset GSE150660, three PLAC8-high immune clusters were characterized, including Cluster 4 (classical monocytes), Cluster 5 (M1-type macrophages) and Cluster 10 (NK cells). Only transcription factors supported by epigenetic evidence from human breast tissue were retained for biological interpretation; monocyte Cluster 4 was enriched with STAT3, NR2F2 and TP53, M1 macrophage Cluster 4 harbored E2F4, while NK cell Cluster 10 showed prominent enrichment of MBD2, PARP1, PML and SIN3A.

Collectively, consistent transcriptional patterns were reproducibly detected across two independent single-cell datasets: epithelial lineage signature transcription factors were generally downregulated, while myeloid immune-related transcriptional regulators were markedly enriched in PLAC8-high monocyte and M1 macrophage subpopulations. This cell-type-specific transcriptional signature implies a potential correlation between PLAC8 expression and the internal transcriptional regulatory network of tumor-infiltrating myeloid immune cells.

**FIGURE 3:**
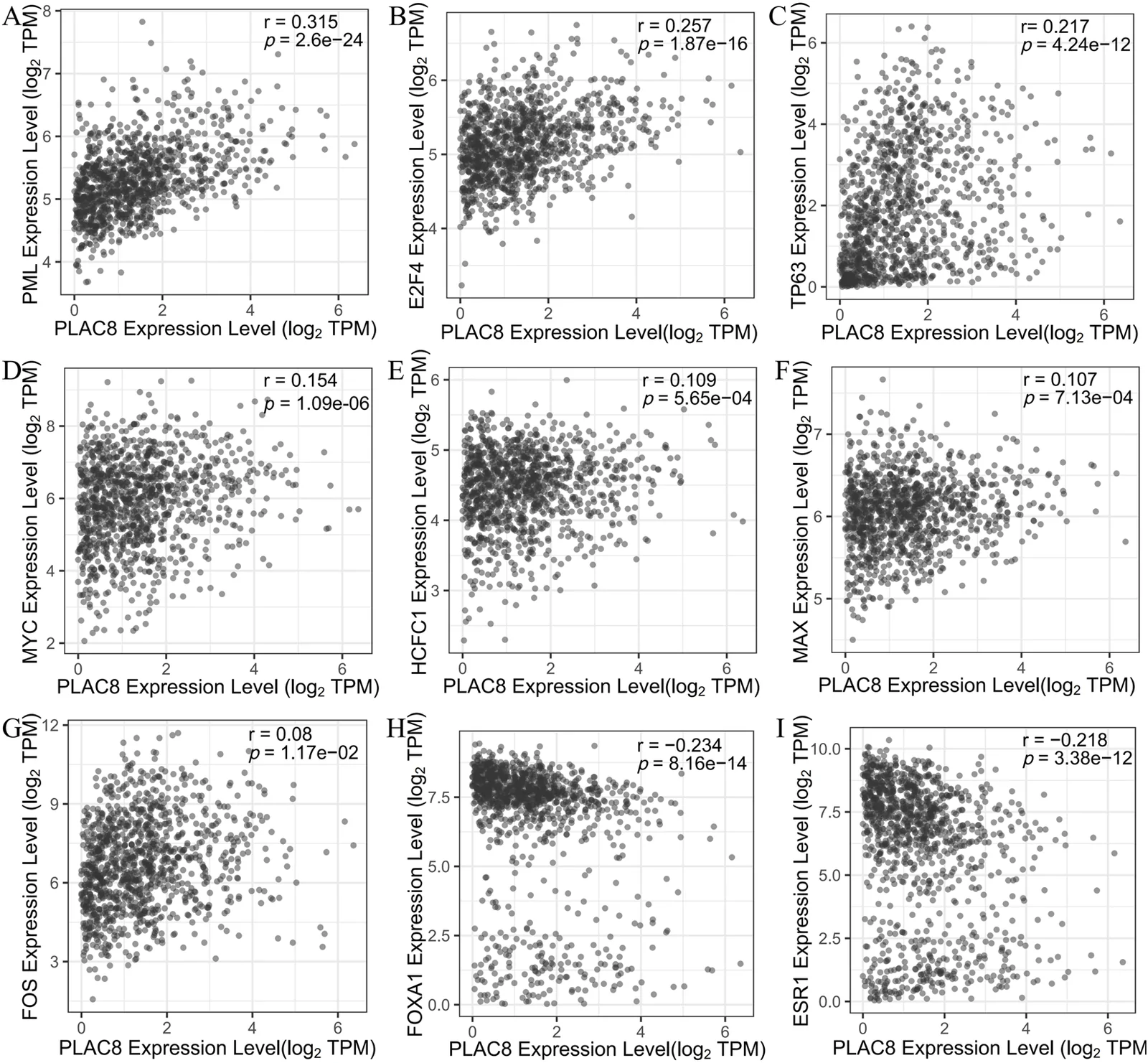
Co-expression correlation between PLAC8 and key transcriptional regulators in breast cancer bulk tissues. Scatter plots display pairwise associations based on log_2_-transformed transcripts per million (TPM) values from the TCGA-BRCA cohort (n = 1,100). Partial Spearman correlation adjusted for tumor purity was adopted as the primary statistical metric to correct bias induced by variable tumor cellularity; unadjusted simple correlation was provided for comparison. **(A–G)** Transcription factors with positive partial correlation with PLAC8: PML, E2F4, TP63, MYC, HCFC1, MAX, FOS. **(H,I)** Transcription factors with negative partial correlation with PLAC8: FOXA1, ESR1. Note: Darker dots represent overlapping individual patient tumor samples.

Bulk-level co-expression profiling based on the TCGA-BRCA cohort (n = 1,100) from TIMER2.0 was performed using partial Spearman correlation adjusted for tumor purity to eliminate confounding signals derived from variable tumor cellularity. Unadjusted simple correlation was also calculated for reference, which confirmed considerable spurious co-expression patterns caused by differential immune and stromal infiltration across tumor samples. Gene co-expression analysis revealed that PLAC8 expression was positively correlated with transcription factors involved in transcriptional control, cell-cycle progression, and cellular stress response, including PML (Figure 3A), E2F4 (Figure 3B), TP63 (Figure 3C), MYC (Figure 3D), HCFC1 (Figure 3E), MAX (Figure 3F), and FOS (Figure 3G). Notably, HCFC1, MAX and FOS exhibited weak correlation magnitudes after purity adjustment and only explained a minor proportion of PLAC8 expression variation. Conversely, PLAC8 expression was negatively correlated with transcription factors linked to breast epithelial differentiation and estrogen signaling, including FOXA1 (Figure 3H) and ESR1 (Figure 3I). All detailed correlation coefficients, 95% CIs, and P values are summarized in Table 1.

**TABLE 1:** Tumor-purity-adjusted partial Spearman co-expression statistics between PLAC8 and selected transcription factors in the TCGA-BRCA cohort (n = 1,100).

Gene symbol	Partial.rho	95% confidence interval	*P* Value	*r* ^2^ (explained variance)	Correlation direction
PML	0.315	0.256–0.371	2.60 × 10^−24^	0.099	Positive
E2F4	0.257	0.197–0.315	1.87 × 10^−16^	0.066	Positive
TP63	0.217	0.156–0.275	4.24 × 10^−12^	0.047	Positive
MYC	0.154	0.091–0.215	1.09 × 10^−6^	0.024	Positive
HCFC1	0.109	0.047–0.169	5.65 × 10^−4^	0.012	Weak positive
MAX	0.107	0.046–0.168	7.13 × 10^−4^	0.011	Weak positive
FOS	0.080	0.018–0.142	1.17 × 10^−2^	0.006	Weak positive
FOXA1	−0.234	−0.293 ∼ −0.173	8.16 × 10^−14^	0.055	Negative
ESR1	−0.218	−0.278 ∼ −0.157	3.38 × 10^−12^	0.048	Negative

Table legend: All co-expression analyses were conducted via the TIMER2.0 platform; partial Spearman correlation (Partial ρ) was used to eliminate confounding bias caused by variable tumor cellularity, while unadjusted simple correlation was calculated as a supplementary control. r2 denotes the coefficient of determination, representing the proportion of total PLAC8 expression variance explained by each paired transcription factor. Weak positive correlation was defined as Partial ρ < 0.1 after purity adjustment, which explained less than 1.5% of PLAC8 transcriptional variation.

These coordinated co-expression signatures reflect a potential linkage between PLAC8 and two distinct lineage-specific transcriptional cascades within bulk breast tumor tissues.

### 3.4 Correlation between PLAC8 expression and immune cell infiltration

**FIGURE 4:**
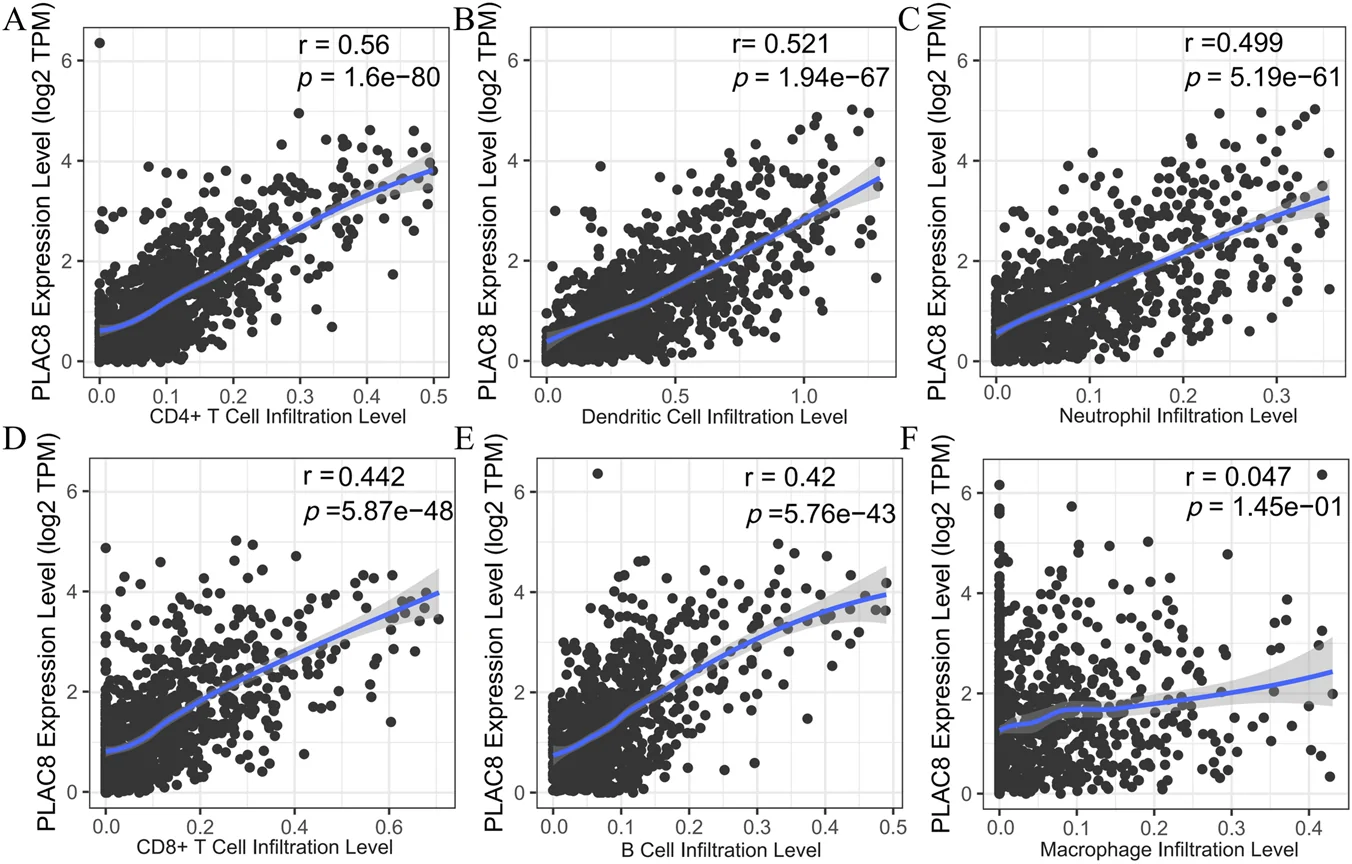
Purity-adjusted partial Spearman correlations between PLAC8 expression and immune cell infiltration in TCGA-BRCA cohort. **(A–E)** Immune cell subsets showing significant positive correlations with PLAC8 expression. **(F)** Immune cell subset showing no significant correlation with PLAC8 expression.

PLAC8 expression showed significant positive purity-adjusted partial Spearman correlations with multiple immune-infiltrating cell subsets including CD4^+^ T cells (r = 0.56, 95% CI = 0.516–0.601, *P* = 1.6 × 10^−80^; Figure 4A), dendritic cells (r = 0.521, 95% CI = 0.474–0.565, *P* = 1.94 × 10^−67^; Figure 4B), neutrophils (r = 0.499, 95% CI = 0.451–0.545, *P* = 5.19 × 10^−61^; Figure 4C), CD8^+^ T cells (r = 0.442, 95% CI = 0.390–0.490, *P* = 5.87 × 10^−48^; Figure 4D), and B lymphocytes (r = 0.420, 95% CI = 0.367–0.469, *P* = 5.76 × 10^−43^; Figure 4E). No significant correlation was observed between PLAC8 expression and macrophage infiltration (r = 0.047, *P* = 0.145; Figure 4F). Collectively, these transcriptomic patterns indicate that PLAC8 does not drive the recruitment and accumulation of immune cells within breast tumor tissue, as evidenced by the non-significant correlation between PLAC8 and macrophage infiltration abundance. Instead, PLAC8 may modulate the functional polarization and phenotypic properties of intratumoral immune populations, particularly macrophages.

### 3.5 Modulation of breast cancer prognosis by interactions between PLAC8 expression and immune cell activity

To independently quantify the intrinsic prognostic value of PLAC8 expression within the TCGA-BRCA cohort, three standardized univariate Cox regression models were implemented: median dichotomization (HR = 0.625, 95% CI = 0.451–0.865, *P* = 0.005); continuous Cox model with PLAC8 treated as a continuous expression variable (HR = 0.874, *P* = 0.002); quartile comparison between the highest and lowest PLAC8 expression subgroups (HR = 0.528, *P* = 0.004). All three analytical approaches consistently indicated that elevated PLAC8 expression serves as a favorable prognostic marker for breast cancer. Four combined subgroups were set to explore synergistic prognostic interactions between PLAC8 and immune infiltration, defined as: 1, low PLAC8 + low immune infiltration; 2, low PLAC8 + high immune infiltration; 3, high PLAC8 + low immune infiltration; 4, high PLAC8 + high immune infiltration. Comparisons of 2 vs. 1 reflect survival benefits of elevated immune infiltration under low PLAC8 background, while 4 vs. 3 quantify prognostic improvements brought by high immune infiltration in patients with high PLAC8 expression.

**FIGURE 5:**
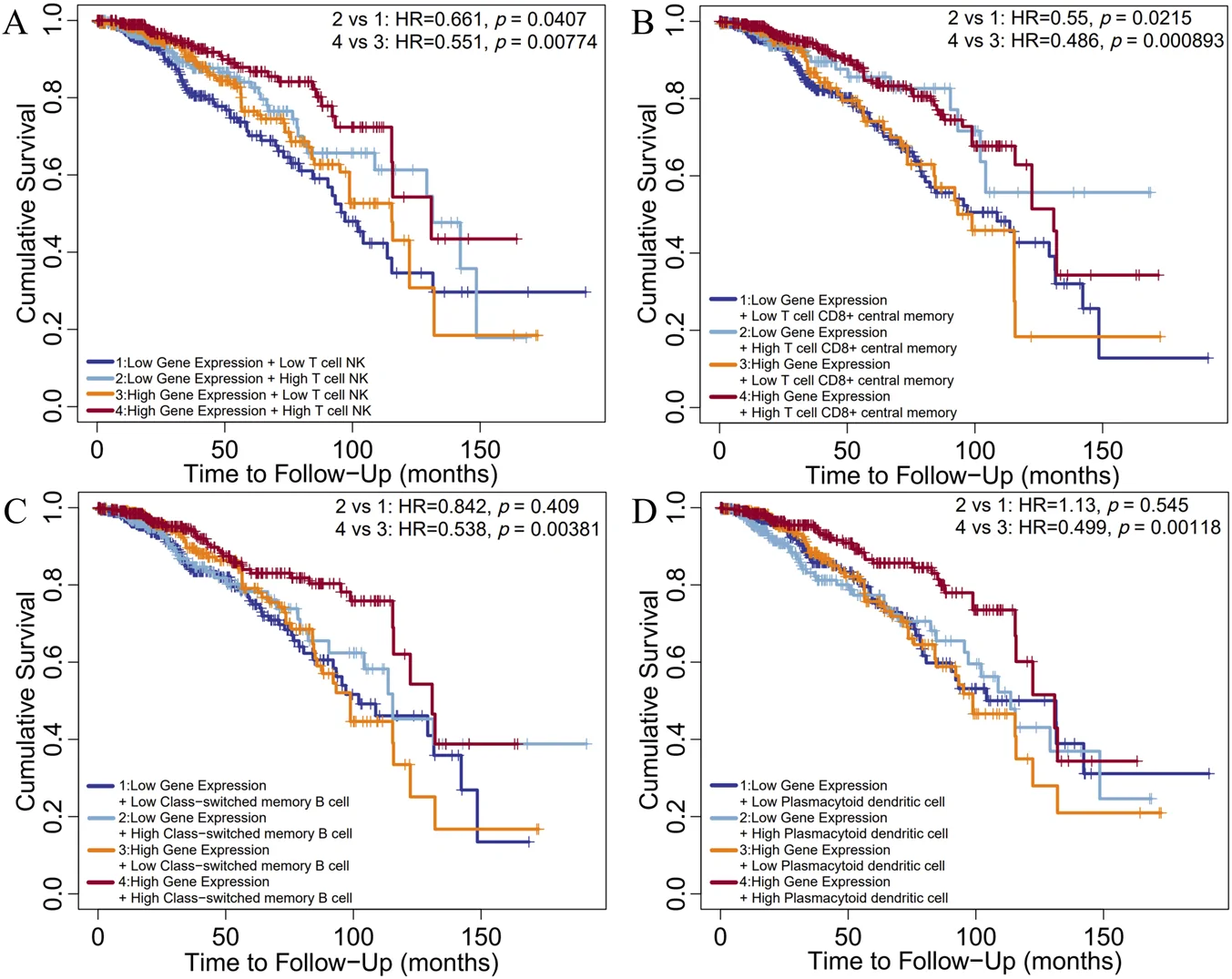
Synergistic prognostic interactions between PLAC8 expression and immune cell infiltration in TCGA-BRCA cohort (n = 1,053). Stratification of PLAC8 expression and immune infiltration relied on default median (50th percentile) cutoffs implemented in TIMER1. Four patient subgroups are strictly defined as follows: 1 = Low PLAC8 expression + Low immune cell infiltration, 2 = Low PLAC8 expression + High immune cell infiltration, 3 = High PLAC8 expression + Low immune cell infiltration, 4 = High PLAC8 expression + High immune cell infiltration. Comparisons labeled “2 vs. 1″represent survival differences of elevated immune infiltration under low PLAC8 background; “4 vs. 3″reflect prognostic improvements induced by high immune infiltration within the high PLAC8 subgroup. **(A)** Combined prognostic effect of PLAC8 expression and natural killer T (NKT) cell infiltration. **(B)** Synergistic prognostic pattern between PLAC8 expression and CD8^+^ central memory T cells. **(C)** Prognostic interaction between PLAC8 expression and class-switched memory B cells. **(D)** Combined prognostic impact of pDC infiltration combined with PLAC8 expression status.

Survival analyses based on the TIMER cohort revealed a pronounced synergistic prognostic pattern between PLAC8 expression and the infiltration of multiple immune cell subsets. Natural killer T (NKT) cells conferred significant overall survival benefits in both PLAC8 low- and high-expression groups (2 vs. 1: Hazard Ratio (HR) = 0.661, 95% CI = 0.445–0.982, *P* = 0.0407; 4 vs. 3: HR = 0.551, 95% CI = 0.355–0.855, *P* = 0.00774; Figure 5A). Multivariate analysis further confirmed NKT-cell infiltration as an independent protective factor (*P* < 0.05), indicating that their antitumor activity is not contingent upon PLAC8 expression and suggesting that NKT cells represent a robust source of immune surveillance in breast cancer. CD8^+^ central memory T cells (CD8^+^ T_CM) demonstrated consistent survival benefits across different PLAC8 expression backgrounds (2 vs. 1: HR = 0.55, 95% CI = 0.33–0.92, *P* = 0.0215; 4 vs. 3: HR = 0.486, 95% CI = 0.32–0.74, *P* = 0.000893; Figure 5B). However, their prognostic effect did not remain independent in multivariate models (*P* > 0.05), implying that the functional relevance of CD8^+^ T_CM may be integrated within broader immune regulatory circuits influenced by PLAC8-mediated signaling. In contrast, the prognostic impact of class-switched memory B cells and plasmacytoid dendritic cells (pDCs) displayed marked PLAC8 dependence. In the PLAC8 low-expression group, neither immune subset provided survival advantage. Conversely, in tumors with high PLAC8 expression, increased infiltration of both cell types significantly prolonged overall survival (B cells: 4 vs. 3, HR = 0.538, 95% CI = 0.354–0.818, *P* = 0.00381; pDCs: 4 vs. 3, HR = 0.499, 95% CI = 0.328–0.759, P = 0.00118; Figures 5C,D). These findings suggest that PLAC8 overexpression may potentiate the antitumor activity of specific immune subsets, thereby enhancing the functional architecture of the tumor microenvironment (TME).

Taken together, the synergistic prognostic patterns between PLAC8 expression and immune cell infiltration exhibit marked heterogeneity. NKT cells function as a stable protective immune subset whose prognostic value is independent of PLAC8 expression. Although CD8^+^ T_CM cells are associated with improved survival, they do not serve as independent prognostic indicators, suggesting that their impact may be shaped by broader PLAC8-regulated immunological networks. In contrast, class-switched memory B cells and pDCs confer significant survival benefits only in the context of high PLAC8 expression, implying that elevated PLAC8 levels may potentiate their functional activation and thereby enhance the immunological fitness of the tumor microenvironment.

Notably, the favorable independent prognostic effect of PLAC8 aligns fully with our single-cell observations that PLAC8 is specifically enriched in intratumoral immune cells: higher PLAC8 expression corresponds to greater overall immune infiltration, which intrinsically predicts superior patient survival. The stratified interaction survival results further clarify that PLAC8 cannot be simply interpreted as a pro-tumor factor driving immune evasion; its primary biological effect lies in remodeling the functional state of co-infiltrated immune cells to modify clinical outcomes.

### 3.6 Interaction landscape of proteins highly expressed in immune-infiltrating cells

**FIGURE 6:**
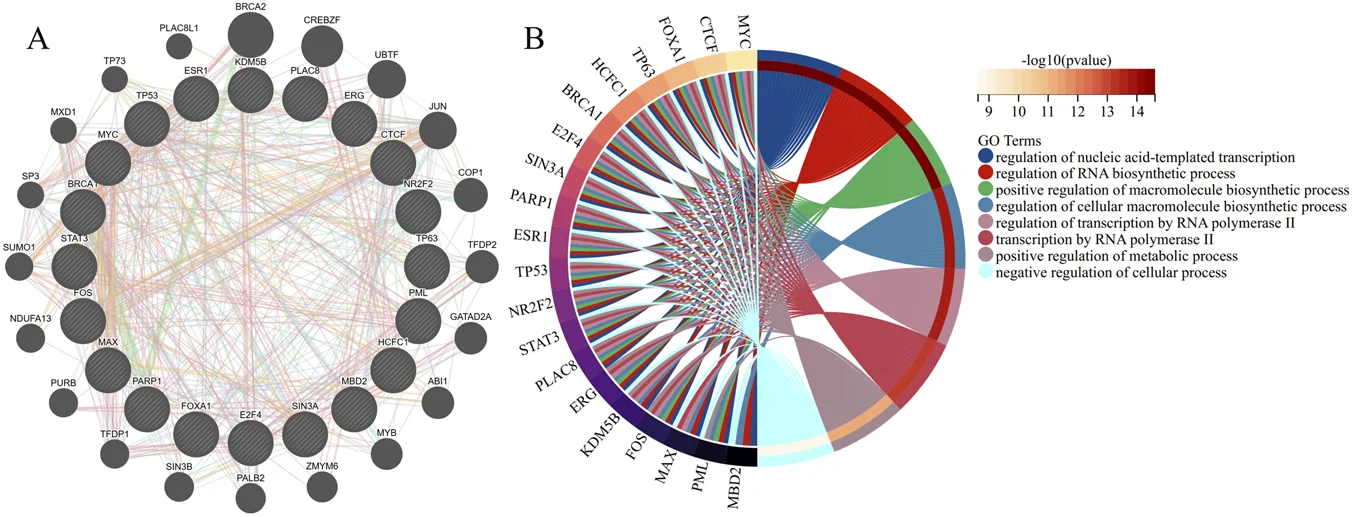
Protein interaction network and functional enrichment analysis associated with PLAC8. **(A)** Core protein–protein interaction network constructed for PLAC8 and immune cell-enriched genes using GeneMANIA. **(B)** Top eight significantly enriched GO biological process terms associated with PLAC8-related genes, ranked by -log_10_(FDR) value after Benjamini–Hochberg multiple testing correction, enrichment performed via SangerBox 3.0.

Using GeneMANIA (version 3.6.0), a multidimensional interaction network was constructed to explore protein–protein relationships between PLAC8 and genes significantly expressed in infiltrating immune cells (Figure 6A). PLAC8 demonstrated direct interactions with FOS, TP53, and MYC, suggesting potential involvement in the MAPK/ERK–MYC axis that drives cell-cycle progression and proliferation, as well as in p53-mediated stress-response and apoptotic pathways. Gene Ontology (GO) enrichment analysis revealed that PLAC8-associated genes were predominantly enriched in pathways related to positive regulation of DNA-templated transcription, regulation of RNA biosynthetic processes, control of macromolecule biosynthesis, and RNA polymerase II–mediated transcriptional regulation. For clarity of visualization, the top eight significantly enriched GO biological process terms (ranked by -log_10_(*P*) value) are presented in Figure 6B. Full statistical results containing exact adjusted FDR values for all enriched GO terms are provided in Supplementary Table S1. These transcriptional and proliferative pathway signatures may mainly reflect the intrinsic functional program of PLAC8 within tumor-infiltrating immune cells, instead of direct oncogenic effects on malignant epithelial cells. Integrating the favorable independent prognostic performance of PLAC8 identified in survival analyses, these observations suggest that PLAC8-mediated transcriptional and biosynthetic regulation may help maintain the antitumor activity of intratumoral immune cells, which could partly explain the favorable clinical outcomes associated with elevated PLAC8 expression.

## 4 Discussion

PLAC8 is a regulatory gene with prominent context-dependent dual biological functions (Mao et al., 2021a). Initially identified within placental transcriptome datasets, accumulated studies have validated its critical roles in embryonic development, adipocyte differentiation, steady-state immune homeostasis, and the pathogenesis of multiple human diseases (Mao et al., 2021a; Mao et al., 2022). Previous tumor-related mechanistic investigations have predominantly focused on its intrinsic function within malignant epithelial cells, as summarized in a comprehensive systematic review of PLAC8 cancer biology (Mao et al., 2021a). A series of cellular experiments have confirmed its oncogenic effects in carcinoma cell lines, while current data also imply its divergent immunomodulatory capacity across distinct cell populations (Mao et al., 2022; Mourtada-Maarabouni et al., 2013).

However, nearly all existing transcriptome-based evidence relies on bulk mixed tumor tissue samples and only characterizes PLAC8 activity in cancer cells, while high-resolution single-cell profiling dissecting PLAC8 expression distribution and immunoregulatory roles across heterogeneous immune subsets in primary and metastatic breast cancer lesions remains scarce (Mao et al., 2021a; Mao et al., 2022).

As a pleiotropic regulatory molecule, PLAC8 participates in multiple core signaling cascades including Akt/MDM2, MAPK, and TGF-β/Smad, exerting context-dependent bidirectional tumor-regulatory effects determined by cell lineage and microenvironmental cues. Published *in vitro* cellular assays consistently verify its oncogenic activity within epithelial tumor cells, while single-cell transcriptional profiling from the present study implies that PLAC8 may mediate programs linked to anti-tumor immunity in intratumoral immune populations. Current evidence further links PLAC8 to programmed cell death, stemness maintenance, immunomodulation, and therapeutic tolerance; nevertheless, its upstream transcriptional regulators and integrated signaling crosstalk networks have not been fully delineated (Mao et al., 2021a). Collectively, these published data highlight the multifaceted biological potential of PLAC8 and underscore the necessity of stratified single-cell research to clarify its cell-type-specific functions, especially its capacity to remodel the heterogeneous tumor immune microenvironment and shape clinical therapeutic responsiveness.

This prominent knowledge gap motivated the present integrated multi-omics study, which systematically delineated cell-type-specific PLAC8 transcriptional profiles and its coupled immune regulatory networks by combining two independent single-cell RNA-seq cohorts of primary and leptomeningeal metastatic breast cancer.

By integrating single-cell transcriptomic profiling and multi-layer bioinformatic pipelines, the current study systematically depicted the compositional heterogeneity of the breast cancer tumor microenvironment across primary and metastatic lesions, and identified PLAC8 as a central transcriptional hub coordinating immune cell functional status within this ecosystem.

Compared with prior bulk-level investigations that average signals across mixed cell populations (Mao et al., 2021a; Mao et al., 2019; Mourtada-Maarabouni et al., 2013), the strength of our work lies in the single-cell-resolved landscape mapping of PLAC8 distribution across all immune and non-immune cellular compartments, with parallel comparison between primary and metastatic tumor specimens, constituting the primary novel contribution of this research.

Single-cell transcriptomic signatures have well established widespread phenotypic heterogeneity among tumor-infiltrating immune cells in breast cancer (Azizi et al., 2018), yet cell-type-restricted PLAC8 expression patterns and its downstream regulatory consequences within this diverse immune compartment have not been comprehensively characterized to date.

We observed prominent lineage-restricted transcription of PLAC8 across both breast cancer cohorts: the gene exhibited remarkably high expression exclusively in B lymphocytes, with only negligible baseline transcription detected in NK cells, CD4^+^ T cells, and monocyte-macrophage lineages. This B-cell-dominant enrichment pattern indicates that PLAC8 may primarily modulate the differentiation and effector activation programs of intratumoral B cells, with limited transcriptional regulatory effects on other myeloid and lymphoid immune subpopulations.

Bulk-level partial Spearman co-expression analyses adjusted for tumor purity revealed significant positive correlations between PLAC8 and multiple core transcriptional regulators governing transcription initiation, cell cycle progression, and cellular stress response. These correlative signatures indicate that PLAC8 occupies a central regulatory node interconnecting transcriptional reprogramming, cell cycle turnover, proliferative signaling, autophagy, and stress adaptation, consistent with previously reported mechanistic observations (Mao et al., 2019; Mourtada-Maarabouni et al., 2013). Critically, prior bulk-based co-expression studies only described global gene correlations in unsorted tumor tissues without resolving immune microenvironment heterogeneity; our single-cell localization data further confirm that this transcriptional coupling network is predominantly enriched within PLAC8-positive immune cells, providing novel evidence for cell-type-specific PLAC8 regulatory programs and expanding current functional understanding of this gene.

Conversely, robust negative partial correlations were detected between PLAC8 and epithelial lineage master transcription factors FOXA1 (r = −0.234; *P* = 8.16 × 10^−14^)and ESR1 (r = −0.218; *P* = 3.38 × 10^−12^), implying that elevated PLAC8 abundance may repress canonical epithelial differentiation programs and antagonize estrogen-dependent signaling cascades. While this regulatory axis could drive a basal/stem-like transcriptional state within malignant cells, single-cell transcriptional quantification verified extremely low PLAC8 transcript abundance in carcinoma populations, suggesting this transcriptional repressive effect predominantly operates within immune compartments rather than tumor epithelial cells. This observation coincides with existing clinical evidence that basal-like breast cancer subtypes typically display disordered immune infiltration and inferior clinical outcomes (Kotsifaki et al., 2025), implying a bidirectional linkage between PLAC8-mediated cellular plasticity and immune microenvironment remodeling.

Protein-protein interaction network analysis further validated direct physical interactions between PLAC8 and FOS, TP53, MYC—core mediators governing proliferative signaling, cellular stress sensing, and apoptotic cascades (Mešky et al., 2020; Chen et al., 2022b; Wagner and Eferl, 2005). Subsequent GO biological process enrichment demonstrated robust enrichment of transcriptional regulation and macromolecular biosynthetic programs among PLAC8-interacting genes. Rather than driving malignant progression of epithelial tumor cells, these transcriptional and metabolic signatures likely reflect intrinsic functional reprogramming events supporting effector immune cell activation; such biosynthetic and transcriptional remodeling is universally required for sustained antitumor activity of tumor-infiltrating immune subsets (Mourtada-Maarabouni et al., 2013; Mešky et al., 2020; Chen et al., 2022b; Wagner and Eferl, 2005). Collectively, these interactome and enrichment results imply that PLAC8 indirectly shapes intratumoral antitumor immunity via core transcriptional and metabolic signaling modules.

Survival stratification analysis revealed significant synergistic prognostic interactions between PLAC8 expression and tumor immune infiltration in breast cancer. Systematic Cox regression analyses with multiple modeling strategies consistently validated PLAC8 upregulation as an independent favorable prognostic factor for overall survival, demonstrating the stable predictive value of this gene for clinical outcomes in breast cancer patients.

Further subgroup stratification according to PLAC8 expression and immune infiltration levels uncovered heterogeneous survival patterns among distinct immune cell populations. NKT cell infiltration exerted persistent tumor-protective effects regardless of PLAC8 transcriptional abundance and was verified as an independent prognostic marker, implying that the intrinsic antitumor surveillance function of NKT cells is not modulated by PLAC8-related signaling cascades. In contrast, the survival advantage linked to CD8^+^ central memory T cells could be observed across all PLAC8 subgroups, yet this protective association lost statistical independence after adjusting for PLAC8 expression, which suggests its clinical benefit is embedded within broader PLAC8-governed immune regulatory circuits.

Notably, the survival benefits mediated by class-switched memory B cells and plasmacytoid dendritic cells were strictly dependent on PLAC8 transcriptional levels. These two immune subsets only conferred prolonged overall survival in patients with high PLAC8 expression, while their infiltration status produced no detectable clinical advantage in low-PLAC8 tumors. This phenomenon indicates that abundant PLAC8 may selectively amplify the antitumor effector functions of B cells and plasmacytoid dendritic cells, reshaping the functional competence of the tumor immune microenvironment. Such PLAC8-dependent immune prognostic heterogeneity has not been previously documented in breast cancer cohorts, highlighting the translational value of combined biomarker stratification integrating PLAC8 expression and immune infiltration signatures.

Collectively, the synergistic prognostic associations between PLAC8 and immune infiltration display prominent heterogeneity. NKT cells represent a stable protective immune population with prognosis-independent function of PLAC8, while CD8^+^ T_CM cells exert survival benefits indirectly via PLAC8-modulated immune circuits. Class-switched memory B cells and pDCs only exert antitumor protective effects under high PLAC8 transcriptional backgrounds, implying PLAC8 may potentiate their immune effector activation and improve overall immune competence within the tumor microenvironment.

Notably, the independent favorable prognostic signature of PLAC8 perfectly aligns with our single-cell expression data demonstrating exclusive PLAC8 enrichment within tumor-infiltrating immune cells: higher PLAC8 expression corresponds to elevated global immune infiltration, which inherently predicts superior patient survival. The stratified survival interaction results further clarify that PLAC8 cannot be simply categorized as a tumor-promoting mediator of immune evasion; its primary biological function centers on remodeling the functional phenotype of co-infiltrated immune populations to alter clinical patient outcomes.

The integrated multi-layer analyses presented herein systematically delineate the hub-like regulatory role of PLAC8 in mediating intercellular crosstalk within the breast cancer tumor microenvironment. By dissecting its coordinated transcriptional networks with master lineage transcription factors and its divergent prognostic associations with distinct immune subpopulations, this study expands the landscape of immunoregulatory signaling and uncovers immune-subset-dependent prognostic heterogeneity in breast malignancy. Furthermore, the robust correlations between PLAC8 expression, immune infiltration profiles, and patient clinical outcomes highlight its translational potential as a novel biomarker for immune subtyping, therapeutic response prediction, and multi-dimensional prognostic stratification in breast cancer clinical practice.

All conclusions of this correlative bioinformatic research remain constrained by inherent analytical limitations, which are comprehensively elaborated in the independent Study Limitations section.

## 5 Study Limitations

This bioinformatic correlation-based research bears several inherent analytical limitations, as summarized below.

1. All findings rely solely on transcriptomic computational correlation analysis without complementary *in vitro* cell functional assays or *in vivo* animal model validation; causal molecular mechanisms cannot be inferred merely from associative omics data.
2. The study combines two separate single-cell RNA-seq cohorts and one bulk TCGA transcriptome dataset generated on disparate sequencing platforms and cell annotation pipelines. Divergent cell subtype classification criteria and data normalization workflows across databases may introduce comparative bias during cross-cohort integrated analysis.
3. The leptomeningeal metastatic single-cell cohort only includes three patients with limited cellular yields, and no bone or lung metastatic breast cancer specimens were incorporated, which limits the generalizability of conclusions regarding metastatic tumor microenvironment remodeling.
4. The composite prognostic signature combining PLAC8 expression and immune infiltration has not been externally validated in an independent large clinical cohort, and its predictive capacity for immunotherapy clinical response remains uncharacterized.
5. This study lacks spatial transcriptomics or immunohistochemical experiments to validate the *in situ* spatial distribution of PLAC8 mRNA and protein within tumor tissue sections.

Future work with expanded independent clinical cohorts and dedicated *in vitro*/*in vivo* functional experiments is warranted to verify and extend the correlative observations reported herein.

## Data Availability

The original contributions presented in the study are included in the article/Supplementary Material, further inquiries can be directed to the corresponding author.
